# Understanding Mass Spectrometry: From Ion Generation to Spectral Interpretation

**DOI:** 10.1002/jms.70078

**Published:** 2026-07-14

**Authors:** Arnold Steckel, Dávid Papp, Gitta Schlosser

**Affiliations:** ^1^ MTA‐ELTE Lendület (Momentum) Ion Mobility Mass Spectrometry Research Group, Department of Analytical Chemistry, Institute of Chemistry ELTE Eötvös Loránd University Budapest Hungary; ^2^ Hevesy György PhD School of Chemistry ELTE Eötvös Loránd University Budapest Hungary

**Keywords:** accurate mass determination, analytical chemistry, compound identification, electrospray ionization, mass spectrometry, tandem mass spectrometry

## Abstract

This tutorial provides a structured introduction to mass spectrometry (MS), with particular emphasis on electrospray ionization (ESI)–based measurements. It is intended to support researchers from chemistry and related disciplines in developing a coherent understanding of MS principles, instrumentation, and data interpretation. The architecture of mass spectrometers is outlined, including ion generation, mass analysis, and detection, together with the fundamental processes by which analytes are converted into gas‐phase ions. Emphasis is placed on practical interpretation of mass spectra, including isotopic pattern analysis, molecular mass and elemental composition determination, and the evaluation of spectral peak characteristics such as resolution. The role of tandem mass spectrometry (MS/MS) in structural elucidation, particularly in the context of ESI‐induced fragmentation pathways, is discussed. Terminology is aligned with IUPAC recommendations to ensure conceptual clarity. Selected illustrative examples demonstrate the integration of theoretical principles with experimental practice in compound identification and analysis.

## 1 Introduction

Mass spectrometry (MS) is an analytical technique in which chemical species are converted into gas‐phase ions and subsequently separated and detected according to their mass and charge properties. From these measurements, molecular masses and elemental compositions can be inferred. MS is a highly sensitive and broadly applicable method [1]. Depending on the analyte, ionization efficiency, and instrument configuration, detection limits may extend to the low attomole or even zeptomole range under optimized experimental conditions [2], typically requiring specialized instrumentation and rigorous control of parameters. MS enables the determination of elemental compositions of small molecules as well as sequence information for peptides, proteins, and nucleic acids, thereby supporting structural elucidation. It is widely applied in fields including pharmaceuticals [3], geochemistry, environmental and nuclear science [4], space science [5], and biochemistry [6].

In MS, ions are separated—typically by electric and/or magnetic fields—according to their *m*/*z* values and subsequently detected. The resulting data are visualized as a mass spectrum, in which relative ion abundance is plotted as a function of *m*/*z*. According to IUPAC recommendations, *m*/*z* denotes the dimensionless quantity obtained by dividing the ion mass (expressed in unified atomic mass units, *u*) by the absolute value of its charge number *z* [7]. The historically used expression “mass‐to‐charge ratio” remains widespread in the literature; however, in a strict SI framework, it would correspond to a quantity with units of kgC^−1^. In this tutorial, the IUPAC‐recommended notation *m*/*z* is used consistently.

The term “mass spectroscopy” should be avoided, as MS does not involve electromagnetic radiation–matter interactions and therefore does not constitute a spectroscopy in the physical sense.

**FIGURE 1:**
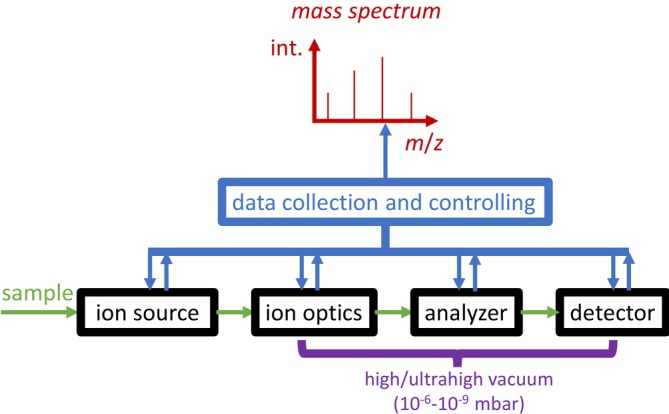
Schematic structure of mass spectrometers.

The schematic design of a mass spectrometer is shown in Figure 1.

The ion source generates gas‐phase ions from the analyte prior to mass analysis. An ion is any atomic, molecular, or radical species with a nonzero net electric charge. In principle, a wide range of samples can be analyzed, provided that their components can be converted into gas‐phase ions by a suitable ionization method. Different ion sources produce different ion types. Table 1 summarizes common ion sources, applications, and typical ions; see also [8].

**TABLE 1:** The most commonly used ionization methods in mass spectrometry and their properties.

Type of ionization	Field of application	Characteristic types of ions formed	Attributes
Electron ionization (EI)	Small, apolar, thermally stable, volatile organic molecules	$M^{+˙}$ (molecular ion)	Hard ionization; molecular ions often fragment in‐source.
Chemical ionization (CI)	Small, apolar, volatile, thermally stable molecules	${\left[M+H\right]}^{+}$ (protonated molecule)	Soft ionization; typically no in‐source fragmentation
Electrospray ionization (ESI)	Small, medium and large‐sized, at least moderately polar, heat‐sensitive molecules	${\left[M+zH\right]}^{z+}$ (protonated molecule); ${\left[M+K\right]}^{+}$, ${\left[M+\mathrm{Na}\right]}^{+}$, ${\left[M+{\mathrm{NH}}_{4}\right]}^{+}$ (cationized molecules); ${\left[M-zH\right]}^{z-}$, (deprotonated molecule); ${\left[M+\mathrm{Cl}\right]}^{-}$, (anionized molecule) etc.	Versatile soft ionization; typically no in‐source fragmentation
Matrix‐assisted laser desorption/ionization (MALDI)	Medium and large‐sized, polar, heat‐sensitive compounds	${\left[M+H\right]}^{+}$, ${\left[M+2H\right]}^{2+}$; ${\left[M-H\right]}^{-}$,	Soft ionization; for peptides, proteins, polymers.

**FIGURE 2:**
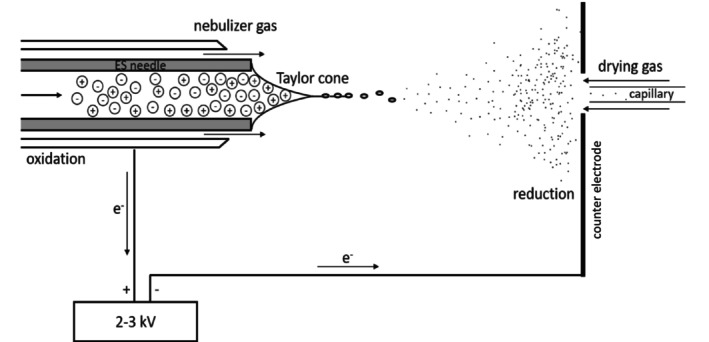
Schematic representation of an electrospray ionization (ESI) ion source.

Electrospray ionization (ESI) is a soft ionization technique widely used in MS for polar and biomolecular analytes. In ESI, the analyte solution is delivered through a capillary held at high electric potential (~2–3 kV) relative to the mass spectrometer inlet. Under the influence of the electric field, the liquid meniscus at the capillary tip becomes deformed into a conical shape known as the Taylor cone [9] (Figure 2). This structure arises from an equilibrium state between electrostatic forces, which pull the liquid outward, and surface tension, which resists deformation.

When the electric field exceeds a critical threshold, a fine liquid jet is emitted from the apex of the Taylor cone and breaks up into a plume of highly charged droplets. As these droplets travel toward the mass spectrometer inlet, solvent evaporation—assisted by a countercurrent drying gas–reduces their size and increases surface charge density.

Droplet stability is governed by the Rayleigh limit, which defines the maximum amount of charge a droplet can sustain before electrostatic repulsion overcomes surface tension. Upon reaching this limit, droplets undergo Coulombic fission, producing smaller offspring droplets that retain a substantial fraction of the original charge. Repeated cycles of evaporation and fission progressively decrease droplet size while concentrating charge, ultimately leading to the formation of gas‐phase ions. This sequence—Taylor cone formation, droplet emission, solvent evaporation, and Coulombic fission—describes the key physicochemical processes governing charged droplet evolution in ESI.

The release of analyte ions from charged droplets is commonly described by two principal models. In the *ion evaporation model (IEM)*, which is most relevant for small ions and low‐mass analytes, ions are emitted directly from the droplet surface once the local electric field exceeds the solvation energy barrier. In contrast, the *charged residue model (CRM)* applies primarily to large, compact biomolecules, such as folded proteins [10]. In this model, droplets undergo successive evaporation and fission events until a single analyte molecule remains, retaining the residual charge of the initial droplet. These models should be regarded as limiting cases of a continuum rather than mutually exclusive mechanisms; their relative contributions depend on analyte size, conformation, and polarity. For flexible or highly extended macromolecules, an intermediate behaviour, often described as chain ejection (*chain ejection model, CEM*), may also occur.

ESI sources can be operated in either positive or negative ion mode, enabling the selective detection of positively or negatively charged ions, respectively. In positive and negative ion modes, ESI often produces multiply charged species, most commonly observed as 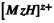 and 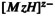. The resulting charge state distribution reflects both intrinsic molecular properties and the physicochemical environment within the droplets. While protonation equilibria are influenced by solution‐phase p*K*_a_ and gas‐phase basicity, the final charge distribution is strongly affected by desolvation kinetics, analyte conformation, and the availability of charge carriers. Large biomolecules can accommodate multiple charges distributed across accessible sites, whereas small molecules are typically detected in singly charged form.

Adduct ion formation is also a characteristic feature of ESI. Species such as 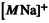, 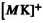, and 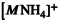 arise when alkali metal cations are present in solvents, buffers, or contaminants compete with protons during ionization. The extent of adduct formation depends on both the concentration of these cations and the analyte's affinity for coordination, for example, through lone electron pairs or polar functional groups.

The role of salts and buffers in ESI is best understood by distinguishing between nonvolatile and volatile components. Nonvolatile salts, such as alkali metal salts or phosphates, do not evaporate during droplet shrinkage and therefore accumulate as solvent is removed. This enrichment increases local ionic strength and the availability of metal cations, promoting adduct formation and reducing the relative abundance of protonated ions. In addition, these species can compete for charge at the droplet surface, contributing to ion suppression [11]. As a result, even low concentrations of nonvolatile salts can significantly distort signal intensities and complicate spectral interpretation. In contrast, volatile buffers, such as ammonium acetate or ammonium formate, are generally preferred in ESI [12] because they are removed during desolvation and do not leave persistent ionic residues. This reduces the likelihood of adduct formation and improves reproducibility. However, at sufficiently high concentrations, even volatile components can contribute to ion suppression through competition for charge, although typically to a lesser extent than nonvolatile salts. Furthermore, certain volatile but highly basic organic modifiers, such as triethylamine (TEA), can induce severe ion suppression even at low concentrations due to their exceptionally high proton affinity.

Solvent composition also plays a critical role in ionization efficiency. High organic solvent content generally accelerates droplet evaporation by lowering surface tension and enhances the transfer of less polar analytes into the gas phase. In contrast, aqueous‐rich conditions slow droplet evaporation and can favor the formation and persistence of multiply charged ions, particularly for large biomolecules such as peptides and proteins.

Finally, matrix effects—particularly ion suppression—represent a major limitation of ESI. Co‐eluting compounds, especially salts and surface‐active species, can alter droplet evaporation dynamics or compete for charge at the droplet interface, leading to decreased ionization efficiency of target analytes. This effect is especially pronounced in complex samples and underscores the importance of appropriate sample preparation and chromatographic separation.

The ions generated in the electrospray source are transferred into the high‐vacuum region of the mass spectrometer for *m*/*z*‐based separation in the mass analyzer. The analyzer type dictates several key performance characteristics of the instrument, including scan range, acquisition speed, spectral peak width (resolution), mass accuracy, and dynamic range. These performance parameters are inherently interdependent and reflect fundamental trade‐offs between resolution, sensitivity, acquisition speed, and dynamic range.

The dynamic range of a mass spectrometer is a key performance parameter. It defines the range of ion abundances that can be detected within a single acquisition, from the noise floor (signal‐to‐noise threshold) to the onset of detector saturation or space‐charge–induced signal distortion. The linear dynamic range (LDR) refers to the subset of this interval over which the detected signal is linearly proportional to analyte concentration with a constant response factor. In practice, the achievable dynamic range depends not only on the mass analyzer and detector but also on ionization efficiency, space‐charge effects, and matrix‐induced ion suppression, particularly in ESI, where competition for charge is often a dominant factor.

Most mass analyzers require (ultra)high vacuum conditions (Figure 1). Modern instruments often contain multiple analyzers—either identical (tandem MS) or different types (hybrid MS)—to combine complementary performance characteristics. Soft ionization typically produces intact ions that provide molecular mass information but limited structural detail; structural elucidation is therefore commonly achieved by gas‐phase ion activation and fragmentation prior to detection (see MS/MS).

Table 2 summarizes the main types of mass analyzers together with their characteristic performance parameters, including resolution, mass accuracy, and typical dynamic range (see also [8].

**TABLE 2:** The type and properties of the most frequently used analyzers in modern mass spectrometry.

Type of analyzer	Resolution	Mass accuracy	Dynamic range (typical)	Principle of operation	Notes
Quadrupole (Q)	~1 k	~0.1–0.5	10^5^–10^6^	Separation using a combination of DC and AC currents coupled to two pairs of cylindrical metal rods	Fast; also used as RF‐only collision cell (MS/MS)
Quadrupole ion trap (QIT)	~1–6 k	~0.05–0.15	10^3^–10^4^	Separation in a 3D quadrupole field	${\mathrm{MS}}^{n}$ experiments possible
Linear ion trap (LIT)	~6–10 k	~0.02–0.10	10^4^–10^5^	Separation in a 2D quadrupole field	Higher sensitivity and resolution than QIT; ${\mathrm{MS}}^{n}$ option
Time‐of‐flight (TOF)	~10–200 k	~0.005–0.05	10^4^–10^5^	Separation by time of flight in a “field‐free space”	Resolution increased with ion mirrors; often Q‐paired (QTOF)
FT‐Orbitrap	~10–1000 k	~0.001–0.005	10^4^–10^5^	Axial oscillation in electrostatic field, FT signal processing	Widely used in proteomics, often Q‐ or LIT‐paired
FT‐ICR	~100–4000 k	~0.0001–0.002	10^5^–10^6^	Ions in high magnetic field undergo RF excitation, generating FT‐processed image current	Very high cost/maintenance; for special applications

Ions exiting the mass analyzer are typically detected by sophisticated electron multipliers, generating an electrical signal. In contrast, Orbitrap and FT‐ICR analyzers detect ions via image currents within the trapping region, and the signal is processed by Fourier transformation.

## 2 Isotopes and the Mass Spectrum

Even simple monoatomic ions often appear as multiple spectral peaks because most naturally occurring elements exist as multiple isotopes. Understanding this requires revisiting the concept of “mass.” In MS, mass is expressed in unified atomic mass units (u) or daltons (Da), which are numerically identical.

One unified atomic mass unit is 1/12th the mass of a ground‐state carbon‐12 atom at rest (approximately $1.660538921\left(73\right)\cdot 10^{-27}$ kg [7]. Table 3 lists the masses of key subatomic particles [13].

**TABLE 3:** Masses of the proton, neutron, and electron in Da units.

Quantity	Numerical value (u)
Proton mass $\left(m_{p}\right)$	1.00727647
Neutron mass $\left(m_{n}\right)$	1.00866492
Electron mass $\left(m_{e}\right)$	0.00054858

**FIGURE 3:**
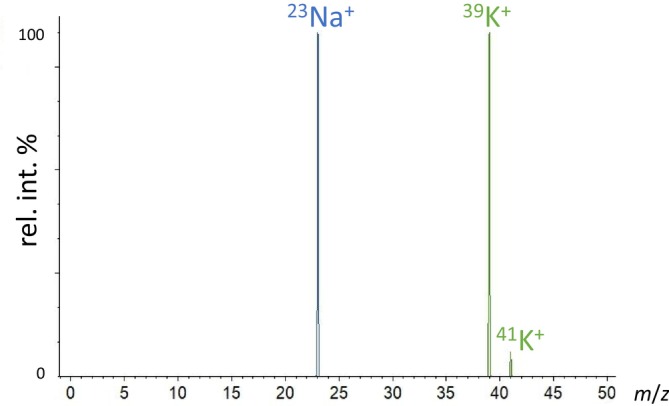
Hypothetical (electron ionization) spectra of Na^+^ (blue peak) and K^+^ (green peaks).

Isotopes are elements with the same atomic number but different mass numbers (due to differing neutron numbers). Most elements possess several stable isotopes. Those existing almost exclusively as a single stable isotope are termed monoisotopic elements (e.g., F, Na, P, I). Simple monoatomic ions (e.g., $A^{+}$ or $A^{+˙}$) of such elements typically produce a single isotopic peak in the mass spectrum (Figure 3, blue peak).

For non‐monoisotopic elements, multiple isotope peaks appear near the expected spectral peak (Figure 3, green peaks); this group of peaks is the ion's characteristic *isotope pattern*. Table 4 [14] lists the natural abundances of common elements in organic chemistry, where “A” denotes the mass number of the most abundant isotope and “A+n” denotes isotopes with n additional neutrons. In this paper, the spectral peak representing the most abundant isotope of a monoatomic ion is consistently designated “A.” Peaks corresponding to its isotopes with one or more additional neutrons are labeled A+1, A+2, etc., while those with fewer neutrons are A−1, A−2, etc.

**TABLE 4:** Isotopes of the most common elements in organic chemistry practice and their abundance.

Element	Isotopes	A%	(A+1)%	(A+2)%
H	^1^H, ^2^H, (^3^H)	99.98	0.02	—
C	^12^C, ^13^C, (^14^C)	98.89	1.11	—
N	^14^N, ^15^N	99.63	0.37	—
O	^16^O, ^17^O, ^18^O	99.76	0.04	0.20
F	^19^F	100.00	—	—
S	^32^S, ^33^S, ^34^S	95.04	0.75	4.20
Cl	^35^Cl, ^37^Cl	75.76	—	24.24
Br	^79^Br, ^81^Br	50.69	—	49.31

**FIGURE 4:**
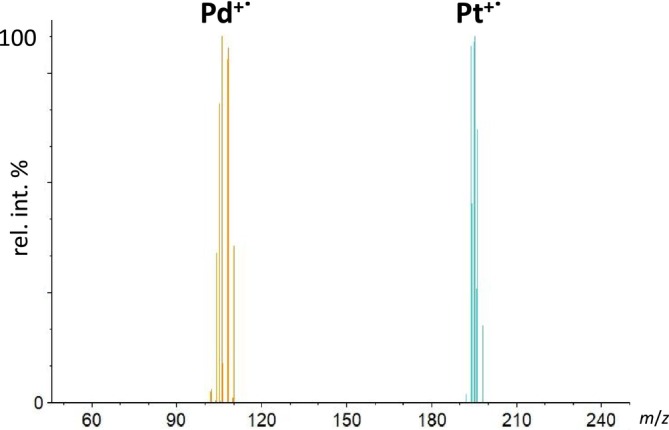
Hypothetical (electron ionization) spectra of Pd^+^˙ (yellow) and Pt^+^˙ (blue). For both ions, isotopic peaks also appear to the left of the most intense peak.

Many metals (e.g., Li, Fe, Pd, Pt, Pb) and some nonmetallic or semimetallic elements (e.g., B, Se, Sn) also have A−n type isotopes (lighter than the most abundant; Figure 4).

For polyatomic ions, isotopic distributions become more complex. In organic compounds, the pattern is predominantly governed by ^13^C abundance. Accordingly, for small molecules (roughly below ~90 carbon atoms) composed mainly of H, C, N, O, and S, the most intense peak within the isotope pattern is often the monoisotopic one. This peak contains only the most abundant isotopes of each constituent element (e.g., ^1^H, ^12^C, ^14^N, ^16^O, ^32^S).

**FIGURE 5:**
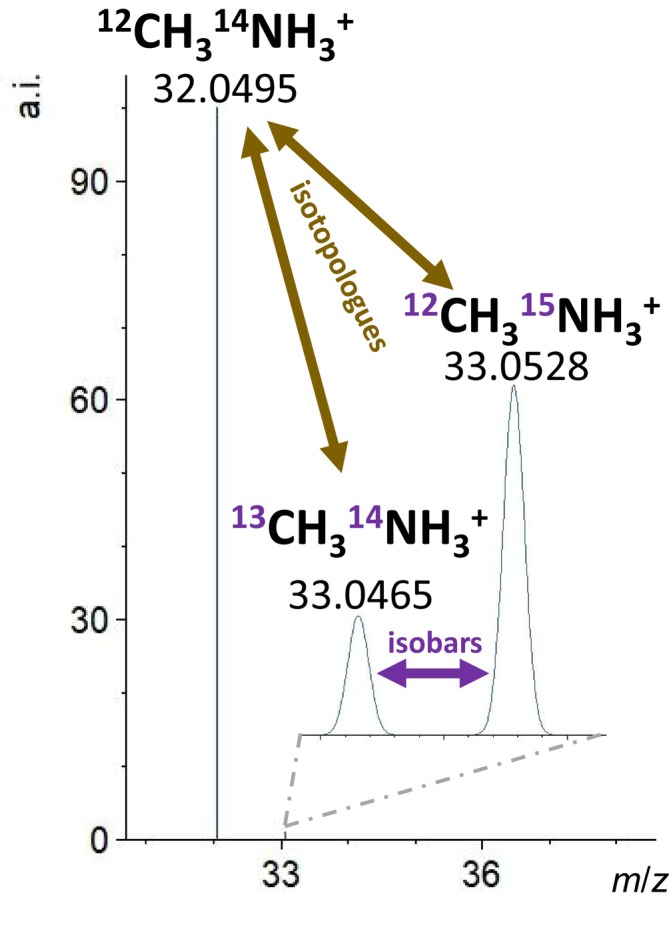
Simulated full isotope pattern of protonated methylamine. The *m*/*z* 32.0495 and 33.0465 are isotopologues of each other, the same is the case for *m*/*z* 32.0495 and 33.0528. The *m*/*z* 33.0565 and 33.0528 are isobars. Although hydrogen isotopes (deuterium and tritium) contribute to the isotope pattern's complexity, their effect is typically negligible; thus, only carbon and nitrogen isotopes are highlighted here.

*Isotopologues* (differing only in isotopic composition [15], e.g., ^12^CH_3_NH_3_^+^ and ^13^CH_3_NH_3_^+^) are located to the right of the monoisotopic peak (Figure 5). Although hydrogen isotopes also contribute to the isotope pattern, their effect is usually negligible; therefore, Figure 5 highlights only carbon and nitrogen isotopes. Isotopologues should not be confused with *isotopomers*, as the latter have the same number of each isotopic atom but differ in their position [16] (e.g., CDH_2_NH_3_^+^ and CH_3_NH_2_D^+^, where D≡H12). MS can distinguish isotopologues based on their different *m*/*z* values, but isotopomers, having identical elemental and isotopic composition, are not distinguishable by MS alone.

Furthermore, a seemingly simple peak may reveal isotopic fine structure with ultrahigh‐resolution MS (i.e., FT‐ICR). For example, zooming in on a CH_3_NH_3_^+^ isotope peak can reveal two distinct peaks due to the very similar *m*/*z* of ^13^CH_3_^14^NH_3_^+^ and ^12^CH_3_^15^NH_3_^+^. Such ions share the same nominal mass but have slightly different exact *m*/*z* values; they are referred to as isobars.

**FIGURE 6:**
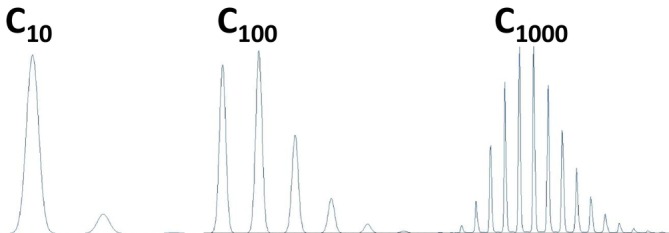
Simulated mass spectra of compounds corresponding to the hypothetical formula C_10_, C_100_, and C_1000_ (considering singly charged species).

As the number of carbon atoms increases, so does the probability of ^13^C incorporation, meaning the base peak may no longer be the monoisotopic peak (Figure 6).

For polyatomic ions, isotopic intensities of individual elements are calculated using the multinomial distribution [17] (Equation 1), and these are then convoluted to obtain the simulated spectrum of the entire ion.

(1) $$p\left(k_{0},k_{1},k_{2},\ldots k_{n}\right)=\frac{N_{X}!}{k_{0}!k_{1}!k_{2}!k_{n}!}p_{0}^{k_{0}}p_{1}^{k_{1}}p_{2}^{k_{2}}p_{n}^{k_{n}}$$

where $N_{X}$ is the number of atoms of element X in the ion, $k_{0},k_{1},k_{2},\ldots k_{n}$ denote the number of atoms of each isotopic form of element X in a given isotopologue, and $p_{0},p_{1},p_{2},p_{n}$ are probability occurrences.

**FIGURE 7:**
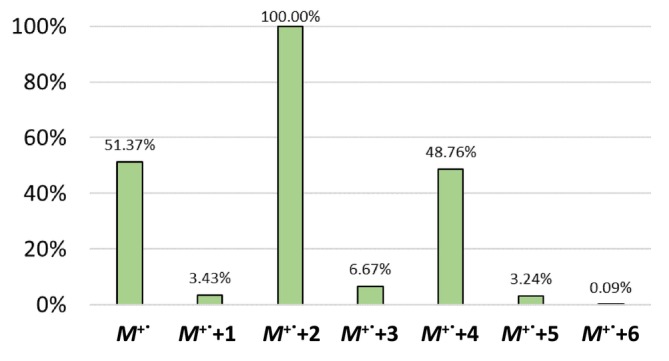
Simulation of the isotope peak distribution of the electron ionization spectrum of 1,2‐dibromobenzene (approximation). The width of the columns has no physical meaning here.

For a practical example, a simulation of the mass spectrum of 1,2‐dibromobenzene $\left(C_{6}H_{4}{\mathrm{Br}}_{2}\right)$ is shown in Figure 7, considering only ^12^C, ^13^C, ^79^Br, and ^81^Br isotopes (neglecting ^2^H effects). Note that in this tutorial, the spectral peak representing the most abundant isotope of a polyatomic ion is consistently designated ‘$M^{+˙}$/M' (molecular ion/protonated molecule). Peaks corresponding to its isotopologues with one or more additional neutrons are labeled $M^{+˙}/M^{\prime }+1$, $M^{+˙}/M^{\prime }+2$, etc., while those with fewer neutrons are $M^{+˙}/M^{\prime }-1$, $M^{+˙}/M^{\prime }-2$, etc. Detailed calculations are in the Supporting Information. For complex spectra or large molecules, approximations like binomial or Poisson distributions are often used. Most MS software includes spectrum simulation capabilities.

While simulations (e.g., for 1,2‐dibromobenzene, Figure 7) are useful for theoretical isotope patterns, ESI spectra need further considerations in practice. ESI typically requires compounds to be at least moderately polar—unlike 1,2‐dibromobenzene, which is an unsuitable analyte for ESI—and spectra can be complicated by adduct ions (e.g., ${\left[M+\mathrm{Na}\right]}^{+}$, ${\left[M+K\right]}^{+}$).

## 3 Resolution and Isotope Spacing

As discussed above, mass spectra can be represented either as profile data (full peak shapes, as exemplified in Figure 6) or as centroided data, in which each peak is reduced to a single *m*/*z*–intensity pair. In routine mass spectrometric data processing, centroiding is typically performed automatically by the instrument software or downstream processing tools, using peak detection and signal fitting algorithms (often approximated by Gaussian or related functions) [18].

*Resolution* (R), previously mentioned with analyzers, is commonly defined as:

$$R=\frac{m/z}{\Delta \left(m/z\right)}$$

where m/z is the centroid m/z value and $\Delta \left(m/z\right)$ is the peak width at a defined relative intensity, typically 50% of maximum height (FWHM—full width at half maximum). Because instrument resolution is generally *m*/*z*‐dependent, the specific *m*/*z* value at which *R* is reported must always be specified [7].

The spacing of isotopologue peaks within an isotope pattern also contains structurally relevant information. For a simple organic compound M forming ${\left[M+H\right]}^{+}$, higher mass isotopologue peaks arise predominantly from ^13^C incorporation. As a result, adjacent peaks are typically separated by approximately 1.003 Da, corresponding to the mass difference between ^13^C and ^12^C. Because the *x*‐axis in a mass spectrum corresponds to *m*/*z* rather than neutral mass, this spacing scales with charge state; for an ion with charge *z*, the observed spacing is:

d≈1.003/z

where d is the spacing between adjacent isotopologue peaks. Accordingly, isotopic peak spacing provides a direct and practical means of determining the charge state of the ion.

## 4 Calculation of the Molecular Mass

MS determines the molecular mass indirectly because ionization converts neutral molecules into gas‐phase ions via processes such as protonation, adduct formation, or electron loss. In electron ionization (EI), the measured species corresponds to the radical cation *M* ^+•^ formed by a loss of an electron from the neutral molecule. In ESI, multiply protonated species are commonly formed. In this case, the observed *m*/*z* values can be described as follows:

(2) $$m/z=\frac{M+zm_{p}}{z}$$

where *M* is the neutral molecular mass, *z* is the charge state, and *m*_p_ is the mass of a proton (1.00728 Da). Accordingly, multiplication of the measured *m*/*z* value by *z* and subtraction of the total proton mass yields *M*. When the molecular formula is known or hypothesized, direct comparison of calculated and measured *m*/*z* values provides higher accuracy and is generally preferred.

Accurate mass calculation requires distinguishing between different mass definitions. Three commonly used concepts are as follows (for IUPAC definitions see [19]):

- Nominal Mass: mass calculated as the sum of the integer (rounded) masses of the most abundant isotopes of each constituent element. In MS, this mass concept is primarily used for rapid estimations.
- Monoisotopic Mass: mass calculated from the exact masses of the most abundant isotopes of its constituent elements. For organic molecules < ~3000 Da, this is the most commonly used mass definition.
- Average Mass: the isotopic abundance–weighted mean mass of an element or molecule. This is widely used in chemistry and becomes relevant for large biomolecules such as proteins.

Consider caffeine (C_8_H_10_N_4_O_2_), cetirizine (C_21_H_25_ClN_2_O_3_), and human ubiquitin (C_378_H_629_N_105_O_118_S) as examples. Their nominal, monoisotopic, and average masses (Table 5), calculated using the mMass 5.5.0 software [20], illustrate these concepts.

**TABLE 5:** Nominal, monoisotopic, and average mass of caffeine, cetirizine, and ubiquitin.

	*M*_nom_	*M*_mono_	*M*_avg_
Caffeine	194	194.0804	194.1911
Cetirizine	388	388.1554	388.8882
Ubiquitin	8555	8559.6167	8564.7568

For caffeine composed solely of H, C, N, O, the nominal, monoisotopic, and average molecular masses are relatively close, though the differences are significant in MS. In contrast, for chlorinated compounds such as cetirizine, the difference between monoisotopic and average mass is significantly larger due to the comparable natural abundances of ^35^Cl and ^37^Cl. For large biomolecules such as ubiquitin, the cumulative effect of isotopic variation becomes substantial, leading to a measurable separation between monoisotopic and average mass values on the order of several daltons. This effect is governed by multinomial isotopic statistics, most clearly illustrated by carbon: With a natural abundance of ~1.1% for ^13^C, the probability of a 378‐carbon molecule containing exclusively ^12^C is effectively negligible under natural isotopic abundance.

**FIGURE 8:**
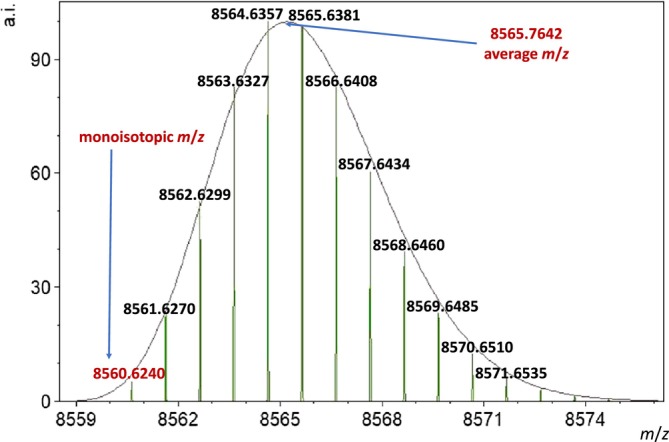
Simulated mass spectrum of a hypothetical, singly protonated molecule of ubiquitin at two resolutions (FWHM = 0.02, green peaks and FWHM = 1.5, brown envelope).

To illustrate, Figure 8 models the hypothetical ${\left[M+H\right]}^{+}$ spectrum of ubiquitin at two resolutions: FWHM = 0.02 (high resolution, green peaks) and FWHM = 1.5 (low resolution, brown envelope). At high resolution, individual isotopologue peaks are resolved and the monoisotopic peak represents a physically well‐defined species. At low resolution, isotopic structure collapses into a single envelope, from which only an average mass can be reliably extracted.

**FIGURE 9:**
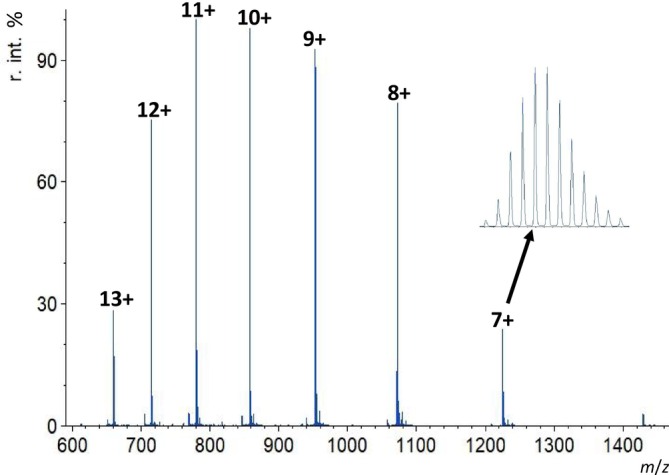
Ubiquitin ESI‐QTOF mass spectrum (Waters Select Series Cyclic IMS), displaying isotopic patterns for multiple charge states (*z*+). Inset: detailed view of the 7+ isotopic pattern.

Ubiquitin can be ionized using soft ionization techniques such as ESI or MALDI. In ESI, the charge state distribution depends not only on molecular mass and basicity but also on solvent composition, additives, analyte polarity, desolvation efficiency, and protein conformation. As a result, ubiquitin is typically observed as a distribution of multiply charged ions, each exhibiting its own isotopic envelope (Figure 9).

Determination of molecular mass from such charge state distributions requires deconvolution. If the charge state can be assigned from isotopic peak spacing, Equation (2) can be applied to each charge state to obtain a set of molecular mass values, which are combined to yield a consensus molecular mass (typically via intensity‐weighted or model‐based approaches). This approach is implemented in most commercial MS software. At lower spectral resolution, additional algebraic constraints may be used to solve simultaneously for charge state and molecular mass, although this requires well‐resolved charge state patterns.

In practice, whether monoisotopic or average mass is reported depends on molecular size and instrument resolving power. For small molecules, monoisotopic mass is typically reported even at unit resolution. For large proteins, the monoisotopic peak is not experimentally observable under natural isotopic abundance, and the reported mass corresponds to the deconvoluted mass of the isotopic envelope.

## 5 Mass Accuracy

Thus far, we have discussed calculated/simulated values. In practice, however, all instruments exhibit measurement errors; therefore, experimentally determined *m*/*z* values must be compared with calculated values (for known compounds). *Absolute mass accuracy/error* is given by:

$$\Delta {\left(m/z\right)}_{\mathrm{abs}}={\left(m/z\right)}_{\exp}-{\left(m/z\right)}_{\text{calc}}$$

where ${\left(m/z\right)}_{\exp}$ is the experimentally observed value, while ${\left(m/z\right)}_{\text{calc}}$ is the calculated value.

Absolute mass error is a signed, dimensionless quantity. Although *m*/*z* is formally dimensionless, small differences are commonly expressed using units such as mDa (and historically mmu) in practical mass spectrometric work. *Relative mass accuracy/error* is obtained by normalizing the absolute error to the calculated value and is typically expressed in parts per million (ppm):

$$\Delta {\left(m/z\right)}_{\mathrm{rel}}=\left(\frac{{\left(m/z\right)}_{\exp}-{\left(m/z\right)}_{\text{calc}}}{{\left(m/z\right)}_{\text{calc}}}\right)10^{6}\left(\mathrm{ppm}\right)$$

In synthetic organic chemistry, high‐resolution MS is typically expected to determine molecular or ion masses within approximately 5 ppm. Because relative error is expressed in ppm, a fixed tolerance corresponds to smaller absolute deviations at low *m*/*z* and larger absolute deviations at high *m*/*z*. Importantly, mass error calculations must always compare corresponding quantities (e.g., an experimentally determined monoisotopic *m*/*z* of a singly protonated ion with its calculated counterpart).

Mass spectrometers equipped with quadrupole‐based analyzers (Q, QIT, LIT; e.g., QqQ or QqLIT hybrids, where q denotes an RF‐only quadrupole) typically provide mass accuracy on the order of 0.1–0.5 Da under routine operating conditions. Instruments incorporating high‐resolution analyzers such as TOF or Orbitrap (e.g., QqTOF, Orbitrap‐based hybrids) routinely achieve mass accuracy in the low parts‐per‐million (ppm) range (typically ≤ 3–5 ppm), depending on *m*/*z*, calibration quality, transient length, and instrument tuning.

Accurate mass determination requires appropriate calibration. In external calibration, reference ions of known *m*/*z* values are measured prior to sample analysis, and a calibration function is generated to relate measured signals (frequencies or flight times) to *m*/*z*. In internal calibration, a reference ion is introduced during data acquisition (often implemented as lock‐mass correction), allowing real‐time compensation for small mass drifts caused by temperature fluctuations, electronic instabilities, or space‐charge effects. The specific implementation depends on instrument design.

In its simplest form, mass recalibration can be described by applying a correction factor:

$$f_{\text{gain}}=\frac{m/z_{\text{calc},\mathrm{ref}}}{m/z_{\exp,\mathrm{ref}}}$$

where $m/z_{\text{calc},\mathrm{ref}}$ is the theoretical value of the reference ion and $m/z_{\exp,\mathrm{ref}}$ is its experimentally measured value. In practice, instrument software applies an appropriate calibration function (which may be linear or higher order, depending on the analyzer) to correct all measured *m*/*z* values across the spectrum.

Continuous internal calibration is not strictly required for all instrument types, but it generally improves long‐term mass accuracy and reproducibility, particularly in high‐resolution measurements.

## 6 Analytical and Structural Information From Mass Spectra

The full mass spectrum offers valuable structural and compound‐type information. The charge state, determined from isotope peak spacing, is influenced by the compound's Lewis acid–base properties with ESI. ESI spectra can be acquired in positive or negative mode, detecting positively or negatively charged species, respectively. In positive mode, Lewis basic compounds readily form ion adducts (e.g., H^+^, Na^+^, K^+^); in negative mode, relatively strong Lewis or Brønsted acids (e.g., sulfonic acids, or less sensitively, carboxylic acids) are detected [21]. Charge state also depends on molecular size: Larger molecules like ubiquitin exhibit wider charge distributions.

Distinctive isotope distributions can indicate specific elements such as Cl, Br, S, or certain metals. In addition to isotopic pattern analysis, the nitrogen rule (Beynon 1960) provides a useful constraint when evaluating singly charged organic ions composed primarily of C, H, N, O, S, P, and halogens. In its classical formulation for odd‐electron molecular ions generated by EI, an odd nominal *m*/*z* value indicates an odd number of nitrogen atoms in the molecular formula, whereas an even nominal *m*/*z* indicates an even (or zero) number of nitrogen atoms. For even‐electron ions such as protonated molecules (${\left[M+H\right]}^{+}$) commonly observed in ESI, the parity relationship is effectively shifted by one mass unit relative to the classical EI formulation.

**FIGURE 10:**
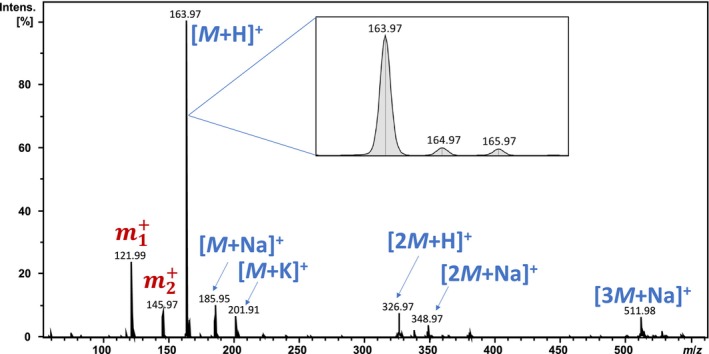
ESI spectrum of *N*‐acetyl‐L‐cysteine in positive mode. The $m_{1}^{+}$ and $m_{2}^{+}$ denote in‐source fragments of the ${\left[M+H\right]}^{+}$ (see Chapter VII).

To demonstrate a systematic interpretation workflow, the positive‐mode ESI spectrum of *N*‐acetyl‐L‐cysteine (NAC) (Figure 10) is evaluated after combining or averaging the relevant part of the so‐called total ion chromatogram (TIC). The TIC represents the sum of all ion intensities recorded at each acquisition time point. The resulting spectrum can be interpreted systematically as follows:

1. Expected Ion Species: In NAC (*M* = C_5_H_9_NO_3_S), no strongly basic functional group is present; protonation in positive ESI therefore primarily involves the carbonyl oxygen atoms of the carboxylic acid and amide functionalities. The amide nitrogen is significantly deactivated due to resonance and does not represent a favorable protonation site. Consequently, the dominant species is typically the protonated molecule ${\left[M+H\right]}^{+}$, with possible alkali adduct formation (${\left[M+\mathrm{Na}\right]}^{+}$, ${\left[M+K\right]}^{+}$).
2. Theoretical *m*/*z* Calculation: The exact *m*/*z* of ${\left[M+H\right]}^{+}$ is calculated (164.0376), which defines the primary target for peak assignment.
3. Protonated Molecule Assignment: The peak at *m*/*z* 163.97 is assigned to ${\left[M+H\right]}^{+}$; since based in Table 2, the difference (~ − 0.07 Da) between the detected and calculated value is well within the typical mass accuracy of a QIT instrument under routine operating conditions. The isotopic spacing of ~1 *m*/*z* confirms a singly charged ion (*z* = 1).
4. Isotopic Pattern Constraint: The *M'* + 2 peak at *m*/*z* 165.97 shows elevated intensity relative to *M'* + 1. For a molecule of this size, this excludes pure CHNO composition and is consistent with one sulfur atom (^34^S contribution, see Table 4).
5. Secondary Peak Assignment: Additional peaks are identified based on the theoretical masses of Na^+^ and K^+^ ions (22.9893 and 38.9632 u, respectively); therefore, *m*/*z* 185.95 and 201.91 are ${\left[M+\mathrm{Na}\right]}^{+}$ and ${\left[M+K\right]}^{+}$, respectively (theoretical values: 186.0195 and 201.9935). The *m*/*z* values of potential oligomeric species can be calculated analogously.
6. Global Consistency: Most major peaks can be explained as adducts, oligomers, of NAC, with no evidence for unrelated components. However, *m*/*z* 121.99 and 145.97 cannot yet be identified. These were identified as in‐source fragments of NAC, as discussed in detail below.

Obviously, this strategy is most effective when the compound is known or can be reasonably constrained a priori.

Accurate mass measurement further refines elemental composition assignment. For small to medium‐sized molecules (typically below ~500 Da), high mass accuracy combined with isotopic pattern constraints and the nitrogen rule substantially reduces the number of plausible molecular formulae. In practice, experimentally determined *m*/*z* values are compared with theoretical values for candidate compositions or database entries. When multiple matches fall within the specified mass tolerance, chemically reasonable restrictions—such as element count limits, H/C ratio, or heteroatom content—are applied to eliminate implausible candidates [22]. Thus, isotopic information, nominal mass parity, and accurate mass jointly contribute to reliable molecular formula determination.

## 7 Multistage MS (MS^2^, MS^*n*^)

Often, molecular formula determination is insufficient due to numerous candidates or, for large molecules (e.g., peptides, proteins), it may be pointless as the number of potential candidates grows exponentially and a single formula can represent many structural isomers. For (bio)polymers, monomer sequence determination is more critical than the molecular formula. Thus, a method providing greater structural insight is needed.

The ion source type significantly influences the ions' energy. For instance, EI generates ions with ~70 eV, enough to break covalent bonds and cause extensive molecular fragmentation [23]. These fragments can be characteristic, aiding identification via database searches, but intense fragmentation can hinder molecular ion identification.

**FIGURE 11:**
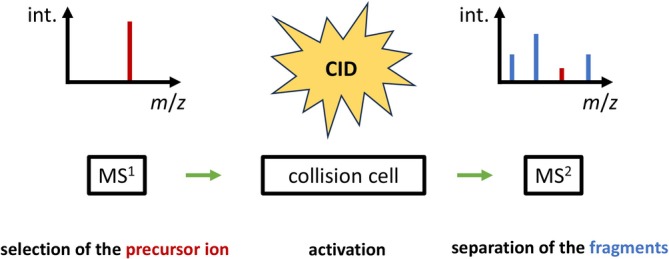
The general scheme of collision‐induced dissociation (CID).

In contrast, ESI is generally considered a soft ionization technique, predominantly yielding intact protonated or deprotonated molecules and adducts with limited fragmentation. However, under certain conditions, substantial ion dissociation may occur within the ion source or ion transfer region due to excess internal energy acquired during ionization or desolvation. This phenomenon, known as in‐source fragmentation, was illustrated previously in Figure 10. To gain structural information from such stable ions, *tandem mass spectrometry (MS/MS* or *MS* ^*2*^ *)* or *multistage mass spectrometry (MS^n^)* is employed. This usually involves selecting a precursor ion population of a specific *m*/*z* with one mass analyzer, inducing its decomposition by excitation (activation) in a dedicated activation region, and then scanning the resulting product ions with another mass analyzer (Figure 11).

During CID, precursor ions are accelerated and collide with neutral gas atoms or molecules (e.g., He, Ar, N_2_), resulting in kinetic‐to‐internal energy transfer. Although energy is deposited via ion–neutral collisions, the subsequent bond cleavage typically proceeds via unimolecular dissociation of an internally energized ion. While this process is conventionally separated spatially across distinct instrument stages (tandem‐in‐space), ion trap instruments achieve MS/MS sequentially within the same physical space (tandem‐in‐time) [24]. This temporal separation uniquely allows for multiple sequential generations of fragmentation (MS^*n*^), where the degree of secondary dissociation can be experimentally controlled through the activation time and energy [24].

Fragmentation follows specific rules. The CID of a singly protonated small molecule (${\left[M+H\right]}^{+}$) in positive ESI mode can be generally described as:

$${\left[M+H\right]}^{+}\to {\left[M+H\right]}^{+*}\to {\left[m+n\right]}^{+}\to m^{+}+n$$

where ${\left[m+n\right]}^{+}$ is a hypothetical ion‐molecule complex, $m^{+}$ is the charged fragment, and n is the neutral fragment molecule, or *neutral loss* (*NL*) [25]. Alternatively, the charge can transfer to n (forming $n^{+}$), leaving m neutral. The relative basicities of m and n determine which carries the charge and which leaves as the neutral molecule: The more basic fragment typically retains the charge. This principle is known as Field's rule. NLs are not directly detected but are inferred from the m/z difference between the precursor and product ions, or between two product ions. Both NLs and fragment ions are important, as they can be characteristic of functional groups. Common neutral losses are listed in Table 6. For a more comprehensive list of NLs and fragments, see [26]. Radical NLs can also occur for some compounds (e.g., those with Cl or Br).

**TABLE 6:** List of some of the most common neutral losses (NLs) in positive mode tandem mass spectrometry.

Mass in *u*	Molecular formula	Observed for
17.0265	NH_3_	Primary amines; analogous NLs (R‐NH_2_) from secondary amines, etc.
18.0106	H_2_O	Alcohols, carboxylic acids, aldehydes, ketones; analogous NLs (ROH) from alkyl ethers and esters
27.9949	CO	Acylium cations (ketones), cyclic ketones
33.9877	H_2_S	Thiol derivatives; analogous NLs (R‐SH) from, for example, thioethers (R‐S‐R)
35.9767	HCl	Chlorine‐containing compounds (also Cl˙)
42.0106	CH_2_CO	Compounds possessing acetyl groups
43.9898	CO_2_	*Aryl* carboxylic acids, anhydrides
46.0055	HCOOH	*Aliphatic* carboxylic acids, analogous NLs (RCOOH) from esters
63.9619	SO_2_	Sulfonic acids, sulfonates, sulfonamides
79.9262	HBr	Bromine‐containing compounds (also Br˙)

Spectra must be interpreted as a whole; fragments must not provide information incompatible with the precursor's composition. In a complex spectrum, a mass difference between two peaks might be coincidental, even with good mass accuracy. Constitutional isomers often fragment to ions with the same *m*/*z* values, but peak intensities (under identical fragmentation parameters, e.g., collision energy) may differ. The N‐rule, previously described, applies to both precursor and fragment ions (see Table 7).

**TABLE 7:** General form of the N rule.

	Odd m/z	Even m/z
Odd‐electron fragment/precursor	Odd # of N	Even # of N
Even‐electron fragment/precursor	Even # of N	Odd # of N

**FIGURE 12:**
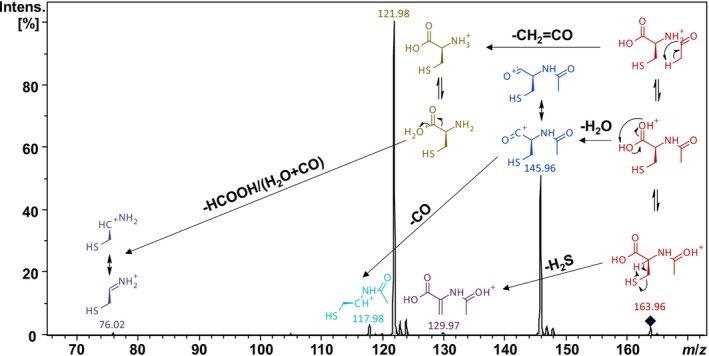
Low‐resolution tandem mass spectrum of *N*‐acetyl‐L‐cysteine acquired on a quadrupole ion trap instrument (Bruker amaZon SL), with assignments of the proposed fragment ions.

As an example, the low‐resolution MS/MS spectrum of the protonated molecule of NAC (Figure 12) is evaluated using a similar stepwise logic as was used above for the full spectrum. (1) Precursor definition: The ion at *m*/*z* 163.96, previously assigned as the protonated molecule, is isolated as the precursor ion. Its low residual intensity in the product ion spectrum (~2.5%) indicates efficient fragmentation under the applied CID conditions. (2) Expected fragmentation behavior: For a small molecule like this without any complex ring structures, typical pathways include neutral losses characteristic of the functional groups present (carboxylic acid, thiol, amide), such as H_2_O, H_2_S, CO, CO_2_, or small neutral molecules derived from the acetyl moiety. (3) Dominant product ion assignment: The base peak at *m*/*z* 121.98 is assigned to ketene (CH_2_CO) loss (Δ*m* ≈ 42 Da) from the precursor ion. This neutral loss is characteristic of acetyl‐containing compounds and is consistent with the presence of the *N*‐acetyl group in NAC. (4) Secondary fragmentation: The ion at *m*/*z* 76.02 is rationalized as a secondary product formed from the *m*/*z* 121.98 ion via subsequent neutral loss of formic acid (HCOOH, Δ*m* ≈ 46 Da), consistent with the presence of a carboxylic acid functionality. (5) Minor product ions: The ion at *m*/*z* 145.96 is consistent with a neutral loss of water (Δ*m* ≈ 18 Da), a common but nonspecific process for oxygen‐containing compounds. The weak signal at *m*/*z* 129.97 is consistent with a loss of H_2_S (Δ*m* ≈ 34 Da), supporting the presence of a thiol group, although its low intensity indicates that this pathway is less favorable under the applied conditions. (6) Global consistency: All major product ions can be explained by chemically reasonable, charge‐directed fragmentation pathways of the protonated NAC molecule. The observed neutral losses (ketene, formic acid, H_2_O, H_2_S) are fully consistent with the known functional group composition, and no fragment ions require the presence of additional components.

Finally, it should be emphasized that the structural information obtainable from low‐resolution MS or MS/MS data alone is inherently limited. In the absence of prior knowledge or suitable constraints, the unambiguous identification of an unknown compound based solely on such data is generally not feasible. Reliable identification therefore requires the combined use of orthogonal information, including chemically reasonable constraints (even minimal prior knowledge about the sample), high‐resolution and accurate‐mass measurements obtained with a properly calibrated instrument, and the joint interpretation of full MS and MS/MS data (with a high‐resolution, accurate‐mass MS/MS spectrum of the model compound *N*‐acetyl‐L‐cysteine included in the Figure DATA S1 as a didactic example illustrating how increased mass accuracy reduces ambiguity in spectral interpretation). In practice, database comparison and manual structure elucidation are used in parallel to arrive at a consistent solution.

Representative fragmentation pathways and general principles for the manual interpretation of tandem mass spectra of small organic molecules are illustrated in our MS/MS tutorial [21]. The same conceptual framework is applied throughout that work to several full MS/MS datasets, including detailed case studies on pharmaceutical compounds.

A fully worked example illustrating this integrated approach for the identification of a completely unknown compound is provided in the Supporting Information of this article, where the workflow is presented step by step.

## 8 Applications of MS/MS

MS/MS significantly enhances the selectivity of analytical methods. QqQ or QqLIT systems, typically coupled with liquid chromatography, are common analytical instruments enabling various fragment analysis (scan) modes (Table 8), all employing CID in a collision cell between two analyzers to induce fragmentation. The CID region is typically an RF‐only quadrupole.

**TABLE 8:** Typical MS/MS fragmentation methods of QqQ and QqLIT [27] mass spectrometers.

Scan type	First analyzer	Collision cell	Second analyzer
Product ion scan	Fixed	Fixed energy CID	Scanning
Precursor ion scan	Scanning		Fixed
Neutral loss scan	Scanning		Scanning
Selected reaction monitoring (SRM)	Fixed		Fixed

The main fragment analysis modes, summarized in Table 8, include the following:

- Product Ion Scan: The first analyzer selects the precursor ion, and the second one scans the resulting product/fragment ions. This method is used for structural analysis and is available on most tandem/hybrid mass spectrometers, not limited to QqQ and QqLIT.
- Precursor Ion Scan: The second analyzer is set to detect a specific fragment ion, while the first analyzer scans for all precursor ions producing this fragment. This is suitable for identifying compounds or compound classes yielding a characteristic fragment.
- Neutral Loss Scan: Both analyzers scan with a fixed mass offset between them. This identifies groups of compounds that produce characteristic neutral losses and is used, for example, in neonatal screening for metabolic diseases.
- Selected Reaction Monitoring (SRM): Widely used for quantitative analysis. The first analyzer selects a specific precursor, and the second monitors one or more of its characteristic fragment ions. Monitoring multiple such transitions during a chromatographic run is termed *multiple reaction monitoring* (MRM); a subtype of SRM and is a common method for quantifying organic metabolites in biological samples like blood or urine.

In modern high‐resolution tandem mass spectrometers (e.g., hybrid quadrupole–time‐of‐flight or quadrupole–Orbitrap instruments), MS/MS data are typically acquired as full fragment ion spectra rather than predefined precursor–product ion transitions. In such systems, quantitative information can be extracted post‐acquisition by selecting specific precursor–fragment ion pairs from the recorded spectra. Consequently, while SRM/MRM remains the method of choice for highly sensitive targeted quantification on QqQ platforms, conceptually similar targeted analyses can be performed on high‐resolution instruments using full‐scan MS/MS data.

In complex samples, an additional level of selectivity is introduced by the strategy used to select precursor ions for fragmentation. In LC–MS‐based workflows, two principal acquisition strategies are commonly employed. In data‐dependent acquisition (DDA), precursor ions are selected for fragmentation based on their instantaneous signal intensity, typically targeting the top *n* most abundant species in each scan cycle (where usually *n* = 3–10). While this approach yields high‐quality MS/MS spectra, it is inherently biased toward abundant components and may exhibit limited reproducibility in complex mixtures.

In contrast, data‐independent acquisition (DIA) systematically fragments all ions within predefined *m*/*z* windows, providing more comprehensive and reproducible coverage at the expense of increased spectral complexity. The resulting multiplexed fragment ion spectra require computational deconvolution for interpretation. These strategies are particularly well established in proteomics but are increasingly applied in other areas of MS [28, 29].

**FIGURE 13:**
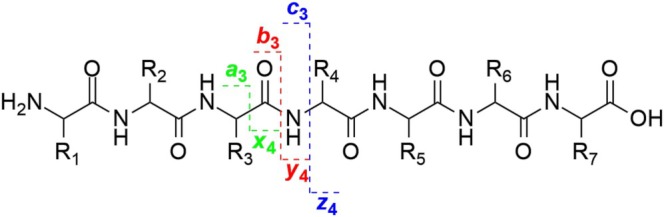
Roepstorff–Biemann–Fohlman nomenclature for naming peptide fragments in MS/MS.

MS/MS, typically implemented using DDA or DIA strategies, is also crucial for identifying and quantifying peptides and proteins [30]. This technique, known as MS/MS‐based proteomics, involves digesting proteins/protein mixtures with specific endoproteinases, followed by reversed‐phase chromatography coupled with MS [31]. Larger peptides typically appear in spectra as multiply protonated molecules. Proton migration during excitation triggers the fragmentation processes. Fragment ions are named according to the Roepstorff–Fohlman–Biemann nomenclature (Figure 13) [32, 33].

**FIGURE 14:**
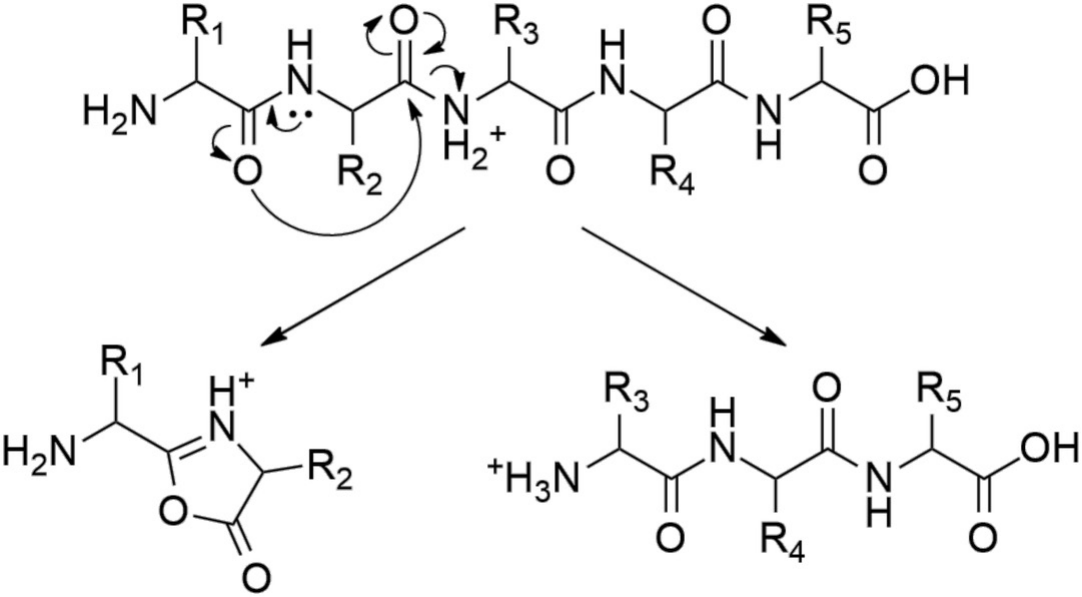
Schematic representation of the most common *b*_*x*_‐*y*_*z*_ peptide fragmentation pathway upon CID.

For *a*‐, *b*‐, and *c*‐ions, indices denote the number of amino acid residues counted from the *N*‐terminus of the original peptide; for *x*‐, *y*‐, and *z*‐ions, counting starts from the *C*‐terminus. Cleavage of amide bonds yields *b*‐ and *y*‐ions, N‐C_α_ bond cleavage generates *c*‐ and $z^{˙}$−ions, and C_α_‐CO bond cleavage produces *a*‐ and *x*‐ions (see Figure 13).^1^ Positive mode CID of peptides predominantly generates *b*‐ and *y*‐ions, typically accompanied by some *a*‐ions. Notably, these bond cleavages are not direct processes but involve complex mechanisms [34]. Here, we briefly discuss the fragmentation pathway to *b*‐ and *y*‐ions, which are primarily used for structure identification (see Figure 14). Although manual interpretation rules are well‐established for peptide fragmentation spectra (e.g., [35]), the vast data from protein mixtures necessitate automated evaluation by dedicated software using database searches.

Beyond proteomics, MS is also extensively applied in other omics disciplines, including metabolomics and lipidomics, highlighting its broad utility in molecular systems analysis; however, a detailed discussion of these areas lies beyond the scope of the present tutorial. Nevertheless, proteomics remains the most methodologically mature and structurally informative example of MS‐based omics workflows.

While the foundational principles and practical workflows detailed in this tutorial are sufficient for a newcomer to master the basics of MS, the applications discussed above represent only a subset of the field's full analytical capabilities. MS is continuously evolving, with a range of specialized methodologies extending its scope into distinct application domains. *Imaging mass spectrometry (MSI)*, for example, enables spatially resolved molecular mapping of drugs, metabolites, and proteins directly on biological tissues, effectively providing a form of molecular histology [36]. *Native mass spectrometry* represents another important extension, allowing the investigation of intact biomolecular complexes in the gas phase while preserving non‐covalent interactions that reflect solution‐phase structure and assembly states [37]. Although these approaches are highly powerful, they are more specialized in scope and are therefore mentioned here only briefly for completeness. In addition, the integration of ion mobility spectrometry with MS (IM–MS) introduces an additional separation dimension based on ion shape and collision cross section, enabling the resolution of isomeric and conformational species that are otherwise indistinguishable by *m*/*z* alone [38]. The ongoing development of such powerful applications further expands the analytical reach of MS and reinforces its central role in modern chemical and biochemical analysis.

## 9 Conclusion

This tutorial provided an introductory overview of the fundamental principles, instrumentation, and analytical applications of MS, with particular emphasis on ESI‐based workflows. The discussion covered ion formation, isotopic pattern interpretation, and MS/MS as a key tool for structural elucidation.

**FIGURE 15:**
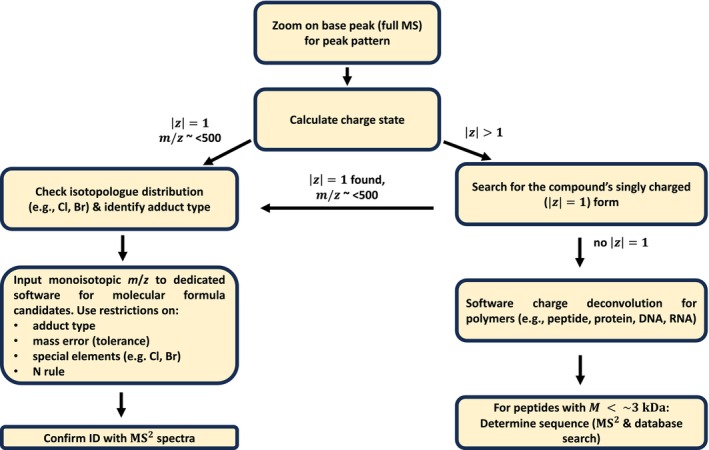
A general workflow for the identification of organic compounds by using mass spectrometry.

A central aspect of mass spectrometric data interpretation is the systematic evaluation of recorded spectra, typically involving (i) initial data inspection and signal processing, (ii) charge state determination, (iii) isotopic pattern analysis for elemental inference and adduct identification, (iv) comparison with theoretical or database‐derived candidate structures, and (v) interpretation of fragmentation pathways obtained from MS/MS experiments. A generalized workflow summarizing these steps is presented in Figure 15, and a worked example is provided in Supporting Information. While the principles described here are broadly applicable across many areas of small‐molecule and (bio)molecular analysis, it should be emphasized that MS is a rapidly evolving field with a wide range of specialized methodologies beyond the scope of this introductory treatment. Nevertheless, the ability to systematically interpret mass spectrometric data remains a fundamental requirement for reliable compound identification and structural characterization across disciplines.

## Author Contributions

**Arnold Steckel** and **Gitta Schlosser** designed the tutorial. **Arnold Steckel** performed the measurements, collected, organized, and interpreted the data. **Dávid Papp** and **Gitta Schlosser** critically reviewed and revised the manuscript. All authors read and approved the final manuscript for publication.

## Funding

This project was supported by the Lendület (Momentum) Program of the Hungarian Academy of Sciences (HAS, Magyar Tudományos Akadémia [MTA]) and by the SNN 148580 project implemented with the support provided by the Ministry of Innovation and Technology of Hungary from the National Research, Development and Innovation Fund (Nemzeti Kutatási, Fejlesztési és Innovaciós Alap), financed under the SNN_24 funding scheme, and by the Slovenian Research and Innovation Agency (ARIS).

## Ethics Statement

The authors have nothing to report.

## Conflicts of Interest

The authors declare no conflicts of interest.

## Supporting information




**DATA S1:** Supporting Information.

## Data Availability

The datasets generated during and/or analyzed during the current study are available as Supporting Information accompanying this publication.

## Endnotes

^1^
Note that $z^{˙}-$ ions are radicals.
