# Supplementary material for: Understanding Mass Spectrometry: From Ion Generation to Spectral Interpretation
Source: J Mass Spectrom. 2026 Jul 14;61(8):e70078. doi: 10.1002/jms.70078 (PMC13366043; doi:10.1002/jms.70078)
Supplement: Supplementary file 1 — DATA S1: Supporting Information. [file JMS-61-e70078-s001.docx]

Supplementary File

**Understanding Mass Spectrometry: From Ion Generation to
Spectral Interpretation**

*Arnold Steckel^1$^, Dávid Papp^1,2¤^, Gitta Schlosser^1×^**

*^1^MTA-ELTE Lendület (Momentum) Ion Mobility Mass Spectrometry Research Group, ELTE Eötvös Loránd University, Institute of Chemistry, Department of Analytical Chemistry, Budapest, Hungary*

*^$^ ORCID ID:* [0000-0002-4423-0399](https://orcid.org/0000-0002-4423-0399)

*^2^Hevesy György PhD School of Chemistry, ELTE Eötvös Loránd University, Budapest, Hungary*

*^¤^ ORCID ID:* [0000-0002-2006-7777](https://orcid.org/0000-0002-2006-7777)

*^×^ ORCID ID:* [0000-0002-7637-7133](https://orcid.org/0000-0002-7637-7133)

**E-mail address:* [*gitta.schlosser@ttk.elte.hu*](mailto:gitta.schlosser@ttk.elte.hu)

*Phone number: 00361 411 6500, mailing address: ELTE Eötvös Loránd University, Budapest, 1117, Pázmány Péter sétány 1/A, Hungary*

**Manual Calculation of a Compound’s Isotope Distribution**

The detailed calculation for modeling the isotope distribution of 1,2-dibromobenzene is as follows:

The molecular formula of 1,2-dibromobenzene is C_6_H_4_Br_2_. We will examine the isotope distribution of the C_6_H_4_Br_2_^+^˙ molecular ion ($M^{+˙}$) formed by electron ionization (EI).

The relative intensity of each isotopologue is determined using the multinomial distribution (**Eq.1**) from the main manuscript), first for bromine (⁷⁹Br, ⁸¹Br) and then for carbon (¹²C, ¹³C) isotopes.

$$p\left( k_{0}, k_{2} \right)=\frac{N_{\mathrm{Br}}!}{k_{0}!k_{2}!}p_{0}^{k_{0}}p_{2}^{k_{2}}$$

For bromine isotopes, where $N_{\mathrm{Br}}$ is the number of bromine atoms (here, 2), $k₀$ is the count of ⁷⁹Br isotopes, $k₂$ is the count of ⁸¹Br isotopes, and $p₀$ and $p₂$ are the natural abundances of ⁷⁹Br (0.507) and ⁸¹Br (0.493) respectively, the probabilities are:

$$p\left( 2, 0 \right)=\frac{2!}{2!0!}{0.507}^{2}{*0.493}^{0}=0.257 (A)$$

$$p\left( 1, 1 \right)=\frac{2!}{1!1!}{0.507}^{1}*{0.493}^{1}=0.500 (A+2)$$

$$p\left( 0, 2 \right)=\frac{2!}{0!2!}{0.507}^{0}*{0.493}^{2}=0.243 (A+4)$$

since each molecule constitutes either 2 ^79^Br, or 1 ^79^Br and 1 ^81^Br, or 2 ^81^Br. The probabilities are calculated in the same way for ^12^C and ^13^C (in this case there are isotopes of type *k*_0_ and *k*_1_ with occurrences of 0.989 and 0.011, respectively). For instance, the probability of finding exactly three ¹³C atoms ($k₁=3$) in a six-carbon system ($p(3,3$)) is very low (approximately 2.75 × 10⁻⁵, which rounds to 0.000 when expressed to three decimal places as in the simplified table below.

| **Type of Isotope Peak** | ***p*(*k*_0_,*k*_1_)** | ***p*** |
| --- | --- | --- |
| *A* | $p(6,0)$ | 0.936 |
| *A*+1 | $p(5,1)$ | 0.062 |
| *A*+2 | $p(4,2)$ | 0.002 |
| *A*+3 | $p(3,3)$ | 0.000 |
| *A*+4 | $p(4,2)$ | 0.000 |
| *A*+5 | $p(5,1)$ | 0.000 |
| *A*+6 | $p(0,6)$ | 0.000 |

An auxiliary table for the convolution is prepared as follows (the internal values are the products of the corresponding bromine and carbon probabilities):


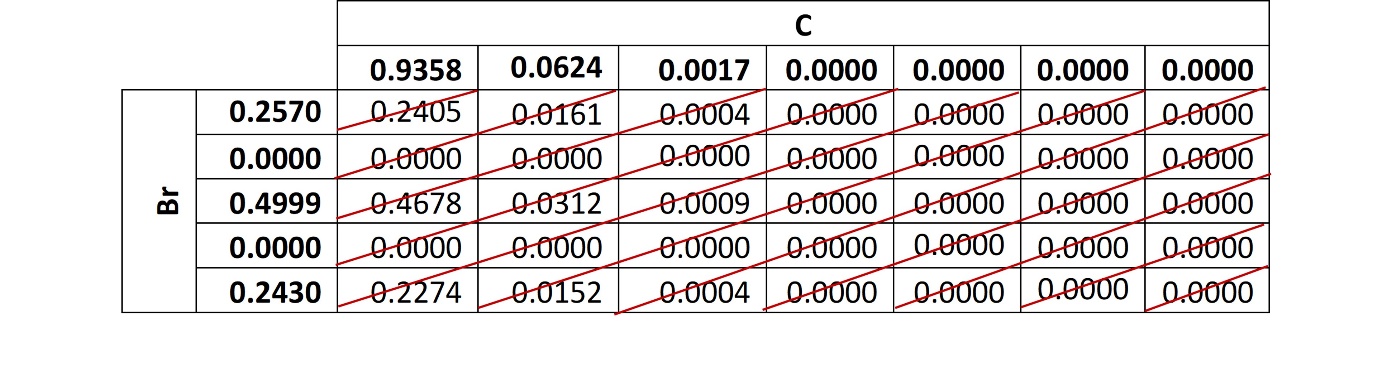


The bromine probabilities are listed for $A$, $A+1$ (contribution is 0), $A+2$, $A+3$ (contribution are 0), and $A+4$ mass shifts relative to the lightest bromine combination.

Next, to obtain the overall isotopic distribution, values are summed along diagonals representing constant total mass shifts (depicted with red lines above). For example:

- **Overall** $\boldsymbol{M}^{\boldsymbol{+˙}}$ **peak intensity:** (C($A$) × Br($A$))
- **Overall** $\boldsymbol{M}^{\boldsymbol{+˙}}\boldsymbol{+1}$ **peak intensity:** (C($A+1$) × Br($A$)) + (C($A$) × Br($A+1$, which is 0 for Br))
- **Overall** $\boldsymbol{M}^{\boldsymbol{+˙}}\boldsymbol{+2}$ **peak intensity:** (C($A+2$) × Br($A$)) + (C($A+1$) × Br($A+1$, which is 0 for Br)) + (C(*A*) × Br($A+2$ for Br))
- **Etc.**

**Demonstration of the Capabilities of High Resolution and Accuracy Mass Spectrometers**

**
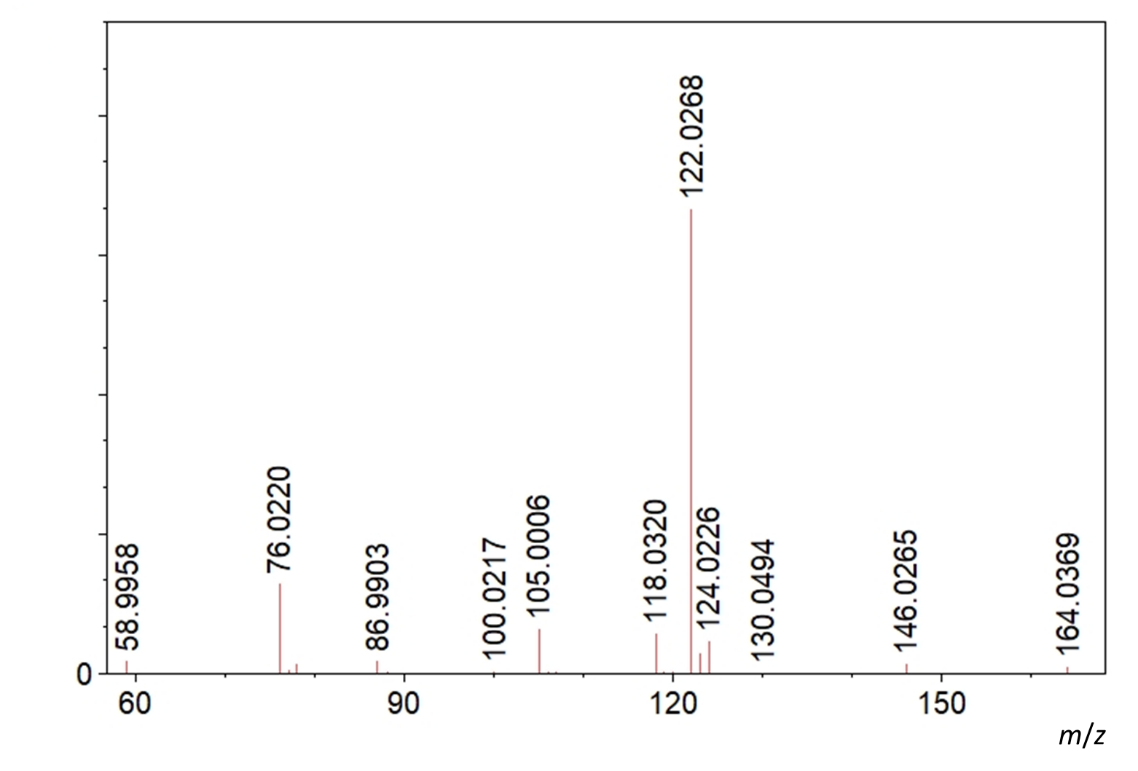
**

**Figure S1** HRAM tandem mass spectrum of *N*-acetylcysteine acquired on a quadrupole-Orbitrap instrument (Thermo Scientific Q-Exactive Focus). The raw data were exported as exact ‘masses’ and visualized by mMass 5.5.0. Notably, the $m/z$ differences for neutral losses in this HRAMS spectrum are significantly more accurate than those obtained from the lower-resolution ion trap spectrum (**Fig. 12** in the main manuscript), facilitating a more unambiguous identification of the neutral losses. For example, an *m*/*z* difference of 33.9875 (between *m*/*z* 164.0369 and 130.0494) is uniquely consistent with a neutral loss of H₂S within the applied mass tolerance to a neutral loss of H_2_S considering a ‘mass tolerance’ of 10 ppm (in this case, the actual difference is -6.5 ppm). The second possible candidate could be H_2_O_2_ with an error of -529 ppm which is too large to be accepted and is also chemically implausible in this fragmentation context. Also, the difference between e.g. *m*/*z* 164.0369 and 122.0268 could only be matched to C_2_H_2_O (ketene, -11.1 ppm) characteristic to compounds containing an acetyl group. The other possible candidate is N_3_ (20.9 ppm) which is very unlikely. For the calculations Chemcalc’s ‘Find molecular formula from a monoisotopic mass’ function was used with the range setting ‘C0-100 H0-200 N0-20 O0-20 F0-3 Cl0-3 Br0-3 S0-2’. The inclusion of chlorine, bromine and fluorine is only for didactic purposes, since the molecule is a known one and does not contain any of these elements.

**Identification of Unknown Compounds Using Mass Spectrometry**

A step-by-step demonstration of the identification of an unknown compound is shown below. The raw spectrum was again acquired on a Q-Orbitrap instrument. The peaks were extracted as exact ‘masses’ (centroiding) using XCalibur 4.1.31.9 and the spectrum was visualized by mMass 5.5.0 (Niedermeyer and Strohalm 2012). The sample was a solution of an unknown pharmaceutical which was diluted 1000x using acetonitrile/water 1:1 (*v*/*v*), containing 0.1% formic acid. The resolution was set to *R*=70,000 and auto source default parameter settings were used for operating the ion source.

**
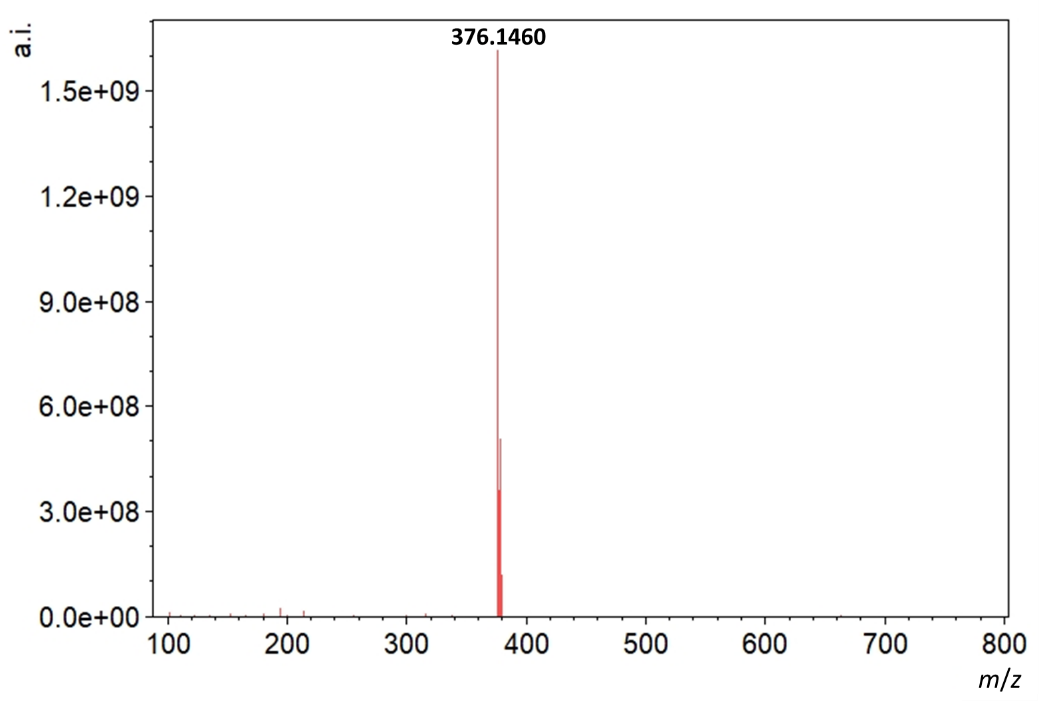
**

**Step 1** Zoom in on the base peak to reveal isotopologue pattern. The zoom-in spectrum is shown below:


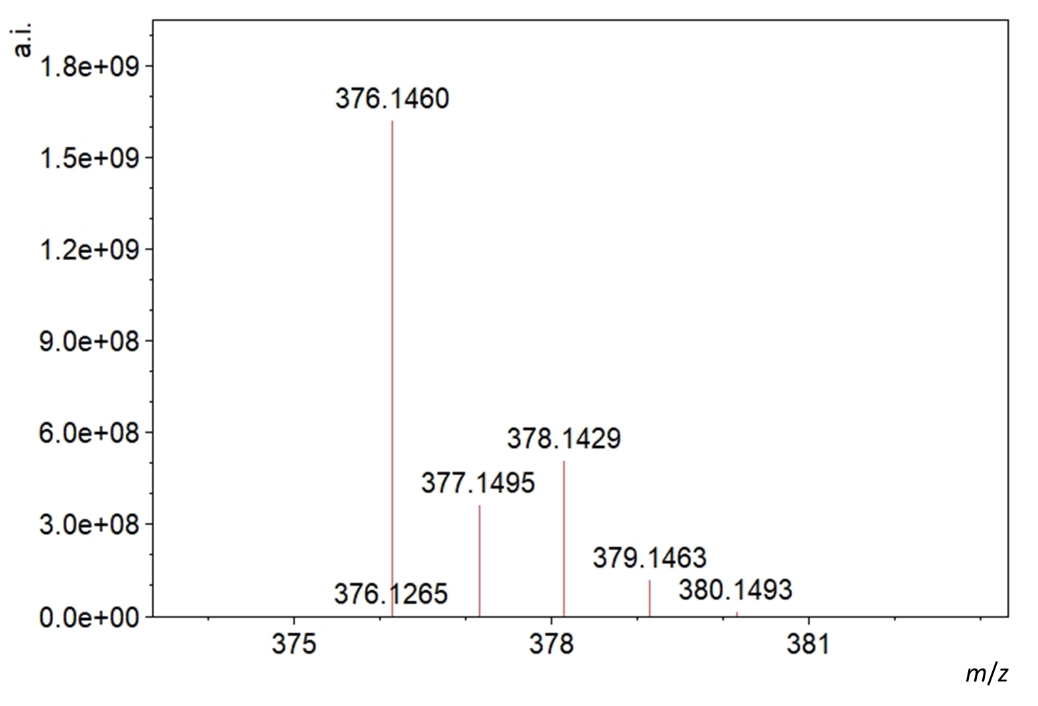


**Step 2** We calculate the charge state of the isotope pattern. The difference between adjacent isotopologue peaks is approximately 1.003 Da, indicating **the ion is singly charged** **and** can be assigned to **an organic compound**. **Suppose** **that the** detected **ion** **is** **a** **protonated molecule**. We can also estimate the molecular mass – keeping in mind that this is just for approximation – which gives us ~375.1387. In that case we can usually detect sodium or potassium adducts further confirming that the peak pattern corresponds to a protonated molecule, but this is not a prerequisite. In this case we could not detect such adducts. As a helpful tool for identifying adducts, the typical *m*/*z* differences are: ~21.9819 for $[{M+Na]}^{+}$ - $[{M+H]}^{+}$ and ~37.9558 for $[{M+K]}^{+}$ - $[{M+H]}^{+}$.

A relatively rough but simple way to estimate how many carbon atoms a small organic isotope pattern contains is to compare the ratio of $M'+1$ to $M'$. In this case this value is $\approx$0.224. Since the occurrence of ^13^C is ~1.1%, the number of carbon atoms should be around 20. This approximation is only valid for small molecules without significant contributions from heteroatoms or halogens. Let us therefore consider the molecule to contain 18-22 carbon atoms just to be sure, no real candidates are excluded in **Step 3**.

The observed isotopologue distribution also differs significantly from that shown for a simple organic molecule in **Fig. 6** (main manuscript), suggesting a 'special element' (e.g., a halogen) is present. The isotopic pattern suggests that this **special element** is **a chlorine**: $M'+2 : M'\approx0.3155$ which is very close to the natural occurrence of chlorine $M'+2 : M'\approx0.3193$.

As demonstrated with dibromobenzene (**Fig. 7**, main manuscript), the isotope pattern changes significantly with the number of halogen atoms. This principle applies to chlorine and other elements with multiple high-abundance isotopes. In such cases, the expected isotopic distribution can be checked by entering the element and its count (e.g., Cl₂, Br₄) into an isotope simulator. For compounds with multiple halogen atoms like Cl₂ or Br₄, the fundamental halogen isotope pattern (e.g., $A'$, $A'+2$, $A'+4$ distributions) will be further convoluted by the carbon isotope contributions. This results in additional peaks ($M'+1$, $M'+3$, etc.) appearing between and around the major peaks derived from the halogen peaks.

**Step 3** By using the information from **Step 2** we can use mMass 5.5.0 ‘Mass to Formula’ function to list the potential candidates which satisfy the above constraints. We type ‘376.1460 to the box ‘Mo. mass’ even though we know that this should be notated as ‘Mo. *m*/*z*’. We specify the charge to be ‘1’ and the tolerance to be ‘5 ppm’. For ‘Minimal formula’ we set ‘C18Cl1’ and for ‘Maximal formula’ ‘C22’. A good advice: always start with stricter constraints and only loosen them if you cannot find any suitable candidates that way. After that, we click on ‘Generate’. The result of our search is shown below:

**
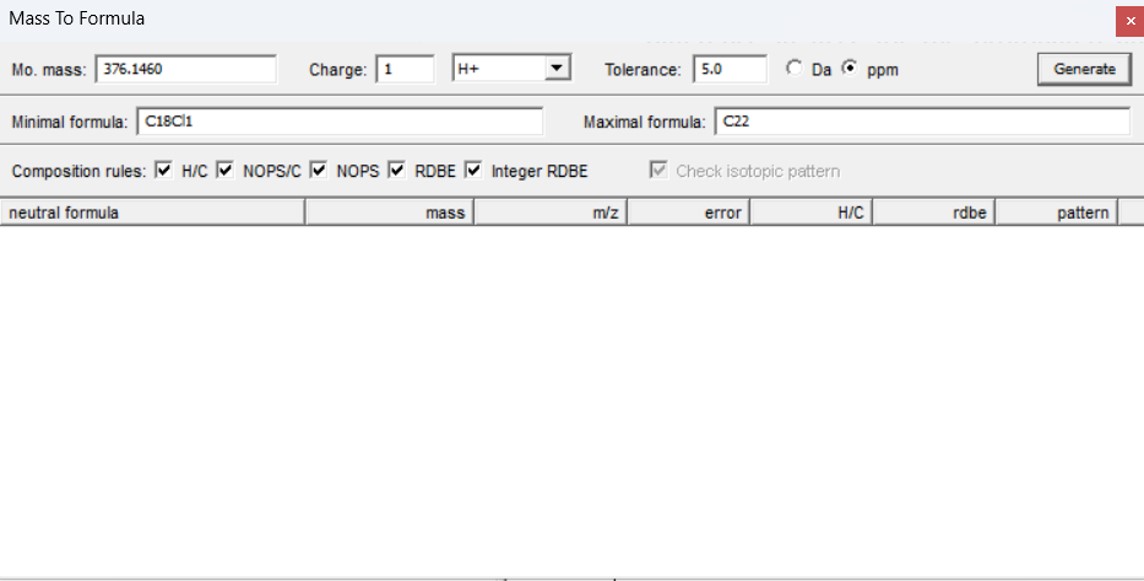
**

Unfortunately, no candidate was found. Knowing that our candidate should be a pharmaceutical we can add further elements in the box ‘Maximal formula’. The most common elements found in organic pharmaceuticals beside H, C, N, O elements are S, halogens and P. Knowing our compound contains one Cl and likely no Br, we expanded the search criteria. Other common heteroatoms in pharmaceuticals—F and I (both monoisotopic), and P (monoisotopic)—were added to the 'Maximal formula' box, allowing for up to three atoms of each. Since sulfur’s isotopic signature can be subtle within a complex pattern, we also included up to three sulfur atoms in the search criteria. After this update we can again run the search by clicking ‘Generate’. This time we get two possible molecular formulae:


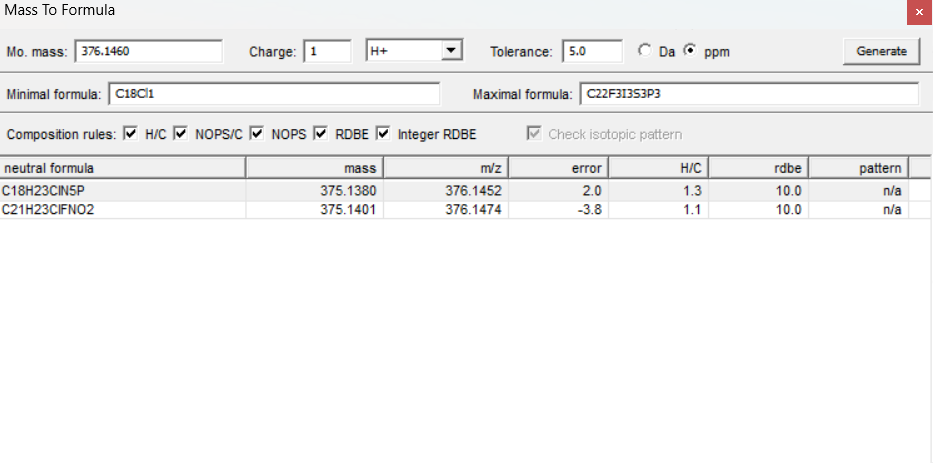


By right clicking on the candidate molecular formula, we can view the isotope distribution or run a ‘Search on PubChem’. Database search yielded candidates consistent with known pharmaceuticals; haloperidol was prioritized based on structural plausibility and MS/MS agreement

**Step 4** We confirm the ID by using the ${MS}^{2}$spectrum. The MS2 spectrum is shown below:


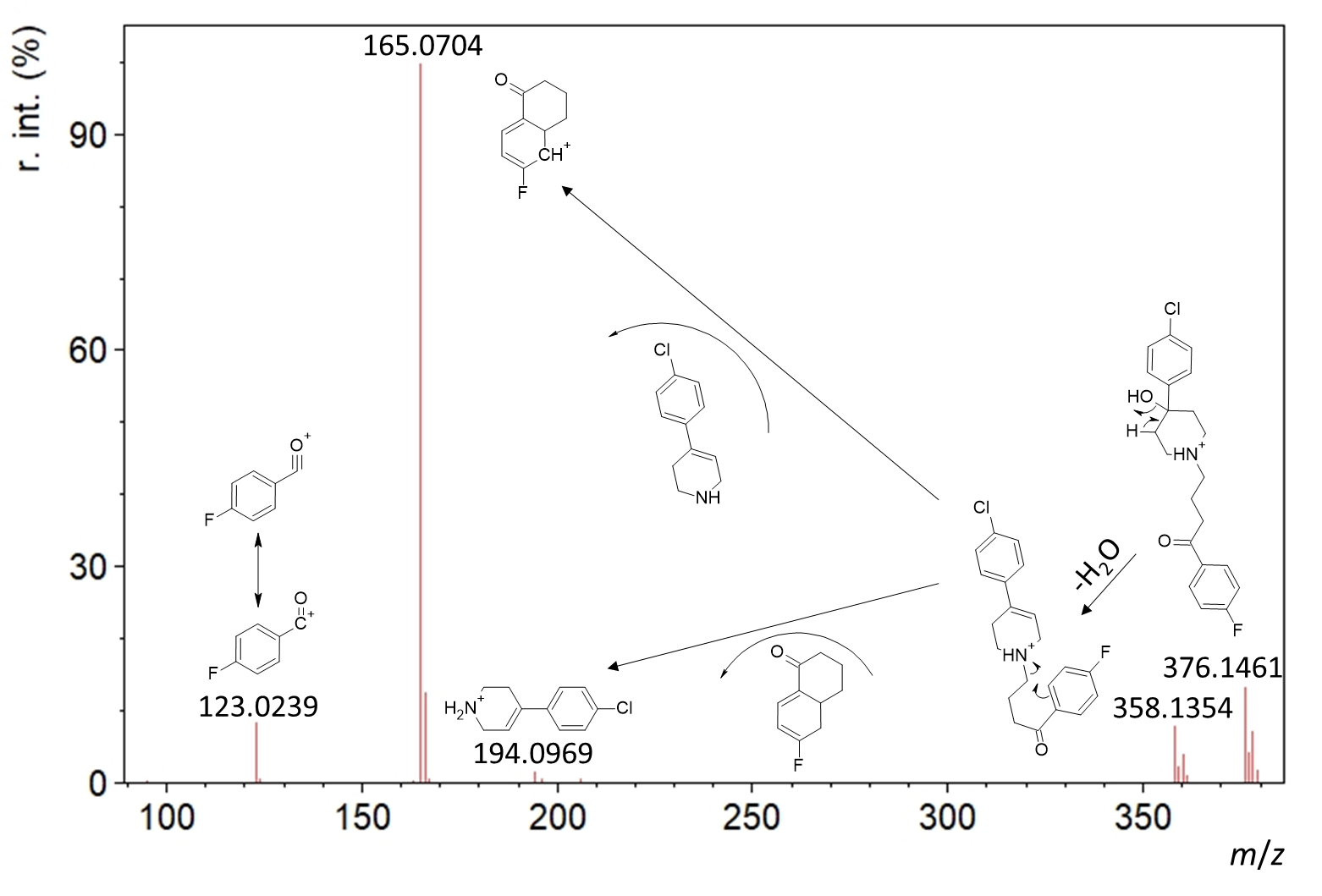


Now we can attempt to confirm the fragments IDs by comparing them with those reported in spectral databases such as MassBank:

<https://massbank.eu/MassBank/RecordDisplay?id=MSBNK-Athens_Univ-AU112202>

Indeed, we can find that the LC-ESI-Q-TOF mass spectrum of haloperidol displays *m*/*z* 376.1402, 358.1297 and 165.0656 matching our expectations. Based on these results, **it is highly likely that the molecule in question was haloperidol.**

And/or we can try to identify possible fragments by ourselves using fragmentation rules of even-electron ions. After being protonated in the source (protonation is expected to occur at the tertiary amine), protonated haloperidol can be cleaved between the protonated nitrogen and the primary carbon linked to it, yielding to a FC_6_H_4_CO(CH_2_)_3_^+^ carbocation (${m/z}_{calc} 165.0710)$. The other high-intensity product ion can be identified as an acylium cation FC_6_H_4_CO^+^ (${m/z}_{calc} 123.0241)$. These acylium type cations are also commonly observed in EI mas spectra as well.

Niedermeyer THJ, Strohalm M (2012) mMass as a Software Tool for the Annotation of Cyclic Peptide Tandem Mass Spectra. PLoS One 7:e44913
